# Supplementary material for: Impact of adult weight management interventions on mental health: a systematic review and meta-analysis protocol
Source: BMJ Open. 2020 Jan 20;10(1):e031857. doi: 10.1136/bmjopen-2019-031857 (PMC7045146; doi:10.1136/bmjopen-2019-031857)
Supplement: Supplementary data [file bmjopen-2019-031857supp002.pdf]

Supplement B: Search Strategy (Medline)

(1) Participants	1.	exp Overweight/ or exp Obesity/ or (adipos* or obes* or over?weight).tw
(2) Weight management intervention	2.	exp Body Weight/ or exp Life Style/ or exp Physical Activity/ or exp Obesity Management/ or exp Diet Therapy/ or exp Exercise/ or exp Diet/ or exp Behavior Therapy/ or exp Health Education/ or ((weight adj3 (body or chang* or los* or maint* or manage* or control* or reduc*)) or (body?mass?index or bmi) or (body adj3 mass) or life?style or (diet* or nutrition*) or (physic* adj3 (activ* or fit*)) or exercis* or (obes* adj3 (intervention or program* or camp* or treat*)) or (behavio?r* or psych*)).tw
(3) Mental health outcomes	3.	exp Behavioral Symptoms/ or exp Emotions/ or exp Mental Disorders/ or exp Adaptation, Psychological/ or exp Mental Health/ or exp Quality of Life/ or exp Self Concept/ OR (depress* or anxiet* or well?being or (quality?of?life or qol or health?status) or (affect* or mood*) or (health?related?quality?of?life or hrqol) or emotion* or (mental adj3 (health or well?being)) or (psych* adj3 (well?being or health)) or self?esteem or self?image or body?image or stress* or (emot* adj3 eating) or binge?eating).tw
(4) Study design	4.	exp Randomized Controlled Trials as Topic/ or randomized controlled trial.pt or controlled clinical trial.mp or randomi?ed.mp or randomly.mp or trial.mp
	5.	1 AND 2 AND 3 AND 4

Limit (5) to English-language results.
