# Supplementary material for: Impact of adult weight management interventions on mental health: a systematic review and meta-analysis protocol
Source: BMJ Open. 2020 Jan 20;10(1):e031857. doi: 10.1136/bmjopen-2019-031857 (PMC7045146; doi:10.1136/bmjopen-2019-031857)
Supplement: Supplementary data [file bmjopen-2019-031857supp003.pdf]

Supplement C: Levels of evidence assessment

Modified flow chart of the decision-making process for levels of evidence - based on study quality and study size. Consistent positive results (66.6% of relevant studies reporting significant positive results) are needed to achieve strong, moderate or limited levels of evidence.

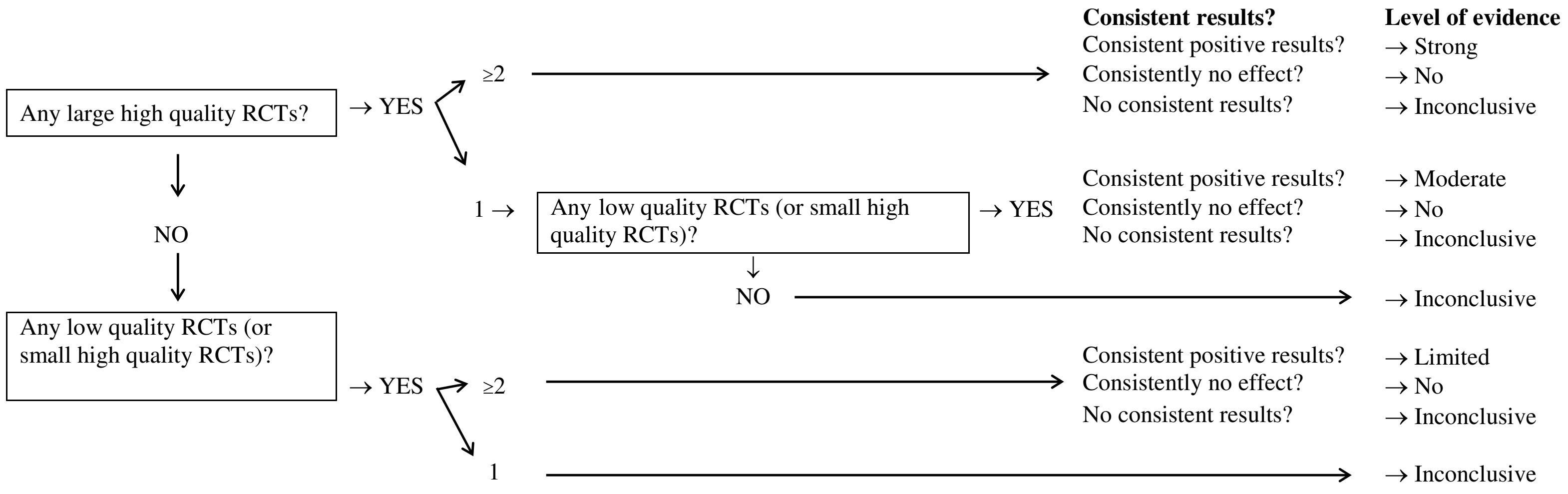

NOTE: studies including ≤ 250 participants or not providing sample size justifying a smaller sample size are considered ‘small’, studies including >250 participants are considered ‘large’.
