# Supplementary material for: Impact of adult weight management interventions on mental health: a systematic review and meta-analysis protocol
Source: BMJ Open. 2020 Jan 20;10(1):e031857. doi: 10.1136/bmjopen-2019-031857 (PMC7045146; doi:10.1136/bmjopen-2019-031857)
Supplement: Author's manuscript [file bmjopen-2019-031857.draft_revisions.pdf]

BMJ Open is committed to open peer review. As part of this commitment we make the peer review history of every article we publish publicly available.

When an article is published we post the peer reviewers' comments and the authors' responses online. We also post the versions of the paper that were used during peer review. These are the versions that the peer review comments apply to.

The versions of the paper that follow are the versions that were submitted during the peer review process. They are not the versions of record or the final published versions. They should not be cited or distributed as the published version of this manuscript.

BMJ Open is an open access journal and the full, final, typeset and author-corrected version of record of the manuscript is available on our site with no access controls, subscription charges or pay-per-view fees (http://bmjopen.bmj.com).

If you have any questions on BMJ Open's open peer review process please email info.bmjopen@bmj.com

BMJ Open

The impact of adult weight management interventions on mental health: a systematic review and meta-analysis protocol.

Journal:	*BMJ Open*
Manuscript ID	bmjopen-2019-031857
Article Type:	Protocol
Date Submitted by the Author:	22-May-2019
Complete List of Authors:	Jones, Rebecca; University of Cambridge, MRC Epidemiology Unit;<br>Lawlor, Emma; MRC Epidemiology Unit, University of Cambridge;<br>Griffin, Simon; The Primary Care Unit, Institute of Public Health<br>van Sluijs, Esther; University of Cambridge, MRC Epidemiology Unit<br>Ahern, Amy; MRC Epidemiology Unit, University of Cambridge
Keywords:	PUBLIC HEALTH, MENTAL HEALTH, EPIDEMIOLOGY

SCHOLARONE™
Manuscripts

I, the Submitting Author has the right to grant and does grant on behalf of all authors of the Work (as defined in the below author licence), an exclusive licence and/or a non-exclusive licence for contributions from authors who are: i) UK Crown employees; ii) where BMJ has agreed a CC-BY licence shall apply, and/or iii) in accordance with the terms applicable for US Federal Government officers or employees acting as part of their official duties; on a worldwide, perpetual, irrevocable, royalty-free basis to BMJ Publishing Group Ltd ("BMJ") its licensees and where the relevant Journal is co-owned by BMJ to the co-owners of the Journal, to publish the Work in this journal and any other BMJ products and to exploit all rights, as set out in our licence.

The Submitting Author accepts and understands that any supply made under these terms is made by BMJ to the Submitting Author unless you are acting as an employee on behalf of your employer or a postgraduate student of an affiliated institution which is paying any applicable article publishing charge ("APC") for Open Access articles. Where the Submitting Author wishes to make the Work available on an Open Access basis (and intends to pay the relevant APC), the terms of reuse of such Open Access shall be governed by a Creative Commons licence – details of these licences and which Creative Commons licence will apply to this Work are set out in our licence referred to above.

Other than as permitted in any relevant BMJ Author's Self Archiving Policies, I confirm this Work has not been accepted for publication elsewhere, is not being considered for publication elsewhere and does not duplicate material already published. I confirm all authors consent to publication of this Work and authorise the granting of this licence.

For peer review only

The impact of adult weight management interventions on mental health: a systematic review and meta-analysis protocol.

Rebecca A. Jones[1], Emma R. Lawlor[1], Simon J. Griffin[1,2], Esther M.F. van Sluijs[1,3], Amy L. Ahern[1]

Corresponding author:

Rebecca A. Jones - rj397@cam.ac.uk / MRC Epidemiology Unit, University of Cambridge, Cambridge, UK.

Author affiliations:

[1] MRC Epidemiology Unit, University of Cambridge, Cambridge, UK.

[2] Primary Care Unit, Institute of Public Health, University of Cambridge, Cambridge, UK.

[3] UKCRC Centre for Diet and Activity Research (CEDAR), University of Cambridge, Cambridge, UK.

Abstract word count: 283/300 words

Full text word count: 3397/4000 words

For peer review only

ABSTRACT

Introduction: The effects of interventions targeting weight loss on physical health are well-described, yet the evidence for mental health is less clear. It is essential to better understand the impact of weight management interventions on mental health to optimise care and minimise risk of harm. We will assess the effect of behavioural weight management interventions on mental health in adults with overweight and obesity.

Methods and analysis: The systematic review will follow the Preferred Reporting Items for Systematic Reviews and Meta-analyses guidance. We will include behavioural weight management interventions with a diet and/or physical activity component focusing on weight loss for adults with a body mass index (BMI) ≥ 25 kg/m^2. Randomised controlled trials (RCT) and cluster RCTs will be the only eligible study designs. Outcomes of interest will be related to mental health. The following databases were searched from inception to 07/05/2019: MEDLINE, Embase, Cochrane database (CENTRAL), PsycINFO, ASSIA, AMED and CINAHL. The search strategy was based on four concepts: (1) Overweight/obese adults defined as ≥18 years, (2) Weight management interventions, (3) Mental health outcomes, and (4) Study design. The search was restricted to English-language published papers, with no other restrictions applied. Two stage screening for eligibility will be completed by two independent reviewers, with two independent reviewers completing data extraction and risk of bias assessment. Data permitting, a random-effects meta-analysis of outcomes, sub-group analyses and meta-regression will be conducted. If not appropriate, narrative synthesis and 'levels of evidence' assessment will be completed.

Ethics and dissemination: Ethical approval is not required as primary data will not be collected. The completed systematic review will be disseminated in a peer-reviewed journal, at conferences and contribute towards the lead author's PhD thesis.

PROSPERO registration number: CRD42019131659

For peer review only

ARTICLE SUMMARY

Strengths and limitations of this study

- The systematic review and meta-analysis will include only randomised controlled trials, offering the highest level of evidence.
- A broad array of mental health outcomes, including mood, stress and depression, will be included in the review.
- A comprehensive search strategy will be used in a large number of databases to maximise the identification of all eligible studies.
- Data permitting, sub-group analysis will be conducted to identify intervention or participant characteristics associated with increased effectiveness.
- High heterogeneity is anticipated across studies which may increase the difficulties in interpreting a meta-analysis.

For peer review only

INTRODUCTION

Rationale

Overweight and obesity is strongly associated with reduced physical health, including a greater risk of cardiovascular disease, type 2 diabetes, stroke, and some cancers (including endometrial, oesophageal and kidney cancer).[1–3] Consequently, individuals with overweight and obesity experience greater all-cause mortality and reduced health-related quality of life.[4,5] Research reports a bidirectional association between obesity and mental health, with those with overweight and obese more likely to have poor mental health and those with mental ill-health at greater risk of weight gain, and consequently, obesity.[6–10] Many have researchers reported improvements in mental health outcomes with weight loss,[11–15] however there has been concern expressed that weight management interventions advocating dietary restriction may contribute to disordered eating and worsen mental health.[16,17] It is essential to better understand the impact of weight management interventions on mental health to optimise care and minimise risk of harm.

Research investigating the relationship between obesity and mental health is increasingly considering mental health as a symptom continuum. The symptom continuum appreciates that individuals can experience one or more symptoms of mental illness without meeting diagnostic criteria for mental illness.[18,19] Considering mental health as a continuum is associated with reduced stigma and improved attitudes towards mental health, highlighting the benefits of broadening the definition of mental health.[19,20] This review will embrace a continuum-based definition of mental health allowing the investigation of a broader range of outcomes from stress, self-efficacy and affect, to symptoms of clinically diagnosed disorders such as depression and anxiety.

While there is clear evidence that weight loss interventions improve physical health, the evidence that they enhance mental health is less clear. Some studies suggest that a focus on weight control can increase stigma and exacerbate symptoms of psychological distress,[21] particularly if goals are not met or if other aspects of life do not change with weight loss.[22] Qualitative research has suggested that there is inadequate support for mental health in obesity management interventions,[23] and a systematic review published in 2014 concluded that weight loss may be associated with improved physical health, but not mental health.[24] Conversely, Fabricatore et al.'s review found statistically significant reductions in depressive symptoms with intentional weight loss trials, although it reported no relationship between weight change and depression,[9] and Lasikiewicz and colleagues' review reported weight management interventions to be associated with improvements in multiple mental health outcomes including self-esteem, body image, quality of life and depressive symptoms.[25]

Previous reviews highlight the breadth of mental health outcomes that could be affected by participation in weight management programmes. However, the majority of reviews focus on a

For peer review only - http://bmjopen.bmj.com/site/about/guidelines.xhtml
For peer review only

limited range of outcomes,[9,16,24–28] and the direction of effects is inconsistent across different outcomes and reviews. It is important to generate a comprehensive understanding of the impact of weight management programmes on mental health as the benefits of improvements in one domain may be undermined by negative impacts on other domains. Previous reviews have also excluded participants with any concurrent disease or clinical psychopathology to constrain the search or to exclude illnesses associated with unintentional weight changes.[16,29] However it is uncommon for an individual with overweight or obesity to be without any concurrent disease or clinical psychopathology due to the greatly increased risk of a wide range of comorbidities,[8] therefore exclusion of these participants limits the representativeness of findings. It is considered beneficial to include participants with comorbid conditions where possible to maximise the generalisability of review findings.

To our knowledge, there is no up-to-date, comprehensive review investigating the effect of weight management interventions on a broad range of mental health outcomes in a representative sample of adults with overweight and obesity. Furthermore, no review has investigated the intervention components most supportive of mental health improvements. Understanding whether intervention components, such as psychological aspects, can attenuate the possible adverse effects to mental health is important for the development of future interventions. This systematic review will apply sub-group analyses and meta-regression techniques to explore the differential effects of intervention or participant characteristics on mental health.

The conflicting findings of previous research and the absence of an up-to-date evaluation of the impact of weight loss interventions on mental health make it difficult to draw clear, reliable conclusions. A comprehensive updated review should increase understanding of the impact of weight management interventions on mental health. The most effective combination of intervention components should be investigated to facilitate improved decision-making in intervention development, aiding the creation of an effective and supportive 'whole-person' intervention.

Objectives

To assess the effectiveness of behavioural weight management interventions compared to minimal, inactive or 'standard care' control groups on mental health in adults with overweight and obesity.

Primary objective*:* (1) Quantify the effect of behavioural weight management interventions on mental health in adults with overweight and obesity.

Secondary objective: (2) Quantify if particular intervention or participant characteristics influence the effect of interventions on mental health.

For peer review only

METHODS AND ANALYSIS

This systematic review protocol adhered to the PRISMA-P reporting guidelines for systematic review and meta-analysis protocols (Supplement A).[30]

Eligibility criteria

Studies will be selected according to the criteria outlined below:

Study designs:

Original peer-reviewed primary research articles reporting randomised controlled trials (RCTs) or cluster RCTs will be included. No restrictions will be placed on year of publication.

Participants:

Participants will be included if they are community-dwelling adults (≥ 18 years old with no upper age limit applied) with overweight or obesity (body mass index (BMI) ≥ 25 kg/m^2) at baseline. Studies that include participants both under and over the age of 18 years will only be included if the data for participants 18 years and older is reported separately. Participants must be seeking intentional weight loss through a behavioural programme. No restrictions will be made on participant demographics. To increase the generalisability of the findings to the general population with overweight and obesity, we will exclude papers that focus exclusively on populations with a physical or mental comorbidity, or pregnant women.

Interventions:

Studies will be included if they evaluated a behavioural weight management intervention that aims to achieve weight loss through changes in diet and/or physical activity. No restriction will be placed on intervention delivery duration, delivery format or on who delivers the intervention. Any study with multiple intervention arms will be included if at least one arm meets the inclusion criteria and separate results are presented for this arm. Interventions aiming to treat eating disorders or involving surgical and/or pharmacological intervention will be excluded.

Comparators:

Studies with a minimal/inactive/standard care control group will be included.

Outcomes:

Included studies are required to have measures of one or more of the following outcomes: Quality of life; Mood/Affect; Stress; Self-esteem; Body image; Emotional eating; Binge eating; Depression;

For peer review only

Anxiety. These a-priori defined outcomes were chosen as they were deemed to be the most relevant and frequently used in previous relevant literature.

Timing:

Defined outcomes must be measured and reported at pre-intervention and at minimum one follow-up point to be eligible for inclusion. The follow-up measurements closest to the time of intervention completion will be extracted for analysis to focus on the immediate intervention effects.

Settings:

Only studies involving participants living in community-based settings will be included.

Language:

Studies published in English language will be included. Non-English language publications will be excluded.

Information sources and search strategy

The following databases were searched from inception to 7th May 2019:

- AMED
- ASSIA
- CINAHL
- Cochrane database (CENTRAL)
- Embase
- MEDLINE
- PsycINFO

Detailed search strategies for each electronic database were developed by RAJ, who has previous experience of conducting systematic reviews, with input from ERL, ALA and a medical librarian. The search strategy contains relevant key words and headings based on previous review articles [25,31–34] and is based on the concepts: (1) Overweight/obese adults AND (2) Weight management interventions AND (3) Mental health outcomes AND (4) Study designs. Terms were adapted from the MEDLINE search accordingly for each database (Supplement B). The search was restricted to English-language papers, with no other restrictions applied. The search strategy was validated through consultation with the systematic review team.

For peer review only

Other resource searches:

To augment the results of the database search, the reference lists of included studies and previous relevant reviews will be searched.[9,16,24–27,29]

Study records

Data management and selection process:

The search results were imported into Covidence systematic review software (Veritas Health Innovation, Melbourne, Australia), and duplications removed. Two researchers initially pilot screened an identical 500 articles to ensure consistency. Any discrepancies in the interpretation of the eligibility criteria were discussed between investigators, with a third reviewer assisting where necessary. Upon completion of pilot screening, the remaining title and abstracts will be independently screened for inclusion by two authors. The full-text of articles identified as potentially relevant will be obtained and dually screened according to the eligibility criteria to ascertain the studies to be included in the review. Eligibility will be discussed for consensus between the two investigators, with a third investigator resolving discrepancies when required. Where necessary, we will seek additional information from study authors to resolve any questions about eligibility. Reasons for exclusion of articles at the full-text screening stage will be recorded. Reviewers will not be blinded to authors, institution or journal when screening articles.

Where studies are reported in more than one publication, all articles will be included and combined to make best use of the data. A Preferred Reporting Items for Systematic Reviews and Meta-Analyses (PRISMA) flow chart will be reported to show the process of study selection.[30]

Data collection process:

Studies meeting the inclusion criteria will have pertinent data extracted using a data extraction form. The data extraction form will be based on the Cochrane data extraction form (2011),[35] the Consolidated Standards of Reporting Trials (CONSORT) statement (2010),[36] and the Cochrane Template for Intervention Description and Replication (TIDieR) [37] to ensure breadth and detail will be captured. The data extraction form will be pilot tested by two investigators on three studies to identify missing or superfluous data items. Independent data extraction will be completed by one investigator with full checking by a minimum of further one investigator. Discrepancies will be resolved through discussion, with use of a third investigator where necessary.

For peer review only

Data items

Data to be extracted will include:

- General information – e.g. study authors, publication year, country, funding source.
- Study details – e.g. study aim, study design, randomisation method, blinding and allocation concealment.
- Participant information – e.g. demographics, recruitment methods, sample size, co-morbidities.
- Attrition/adherence – e.g. total number of participants at baseline and follow up measurements, differential attrition, attendance, study withdrawal, loss to follow-up.
- Intervention information – e.g. setting, content, intervention duration and frequency, profession delivering the intervention, method of delivery, group or individual delivery.
- Comparator information – e.g. setting, content, intervention duration and frequency, profession delivering the intervention, method of delivery, group or individual delivery.
- Outcomes – e.g. mental health outcome(s) studied, whether self-reported or objectively measured, duration of follow-up, statistical analysis, intervention effect sizes.

If a study has multiple arms, data from any arm meeting the inclusion criteria will be extracted where possible. Study authors will be contacted if there are uncertainties regarding the study or missing data.

Outcomes and prioritisation

For all outcomes, prioritisation will be given to units reported as raw data at baseline and post-intervention over data presented as 'mean change' or equivalent. Where possible, data items will be extracted at both study and group level to permit analysis of overall and stratified data (e.g. extracting stratified data to analyse moderation by sex). Study authors will be contacted to request any data required that is not available.

Risk of bias in individual studies

Risk of bias will be independently appraised by a minimum of two review authors. Discrepancies will be discussed between authors for a consensus and a third investigator will be consulted where required.

For peer review only

The Cochrane 'Risk of bias' tool (RoB) will be used to assess the risk of bias in the included studies.[38] The tool assesses the following study features as 'low risk', 'high risk' or 'unclear': (1) Random sequence generation, (2) Allocation concealment, (3) Blinding of participants and personnel, (4) Blinding of outcome assessment, (5) Incomplete outcome data, and (6) Selective reporting.

Other potential sources of bias not covered by the tool will be noted by review authors. Review authors will not be blinded to the included study's information (author names, journal of publication, affiliated institute). A risk of bias graph and summary table will be presented.

Data synthesis

When the data permits, outcome data will be synthesised using a random-effects meta-analysis (Review Manager v5.3, Cochrane Collaboration) due to the predicted diverse range of population and intervention types. Meta-analysis will be conducted on the outcome measures reported closest to the time of intervention completion, regardless of intervention duration, to focus analysis on the immediate intervention effects.

As it is likely a range of outcome measures will be identified, standardised mean difference (SMD) will be calculated. SMD will be categorised using thresholds as small (0.2), medium (0.5) and large (0.8).[39] Where possible, mean differences (for continuous data) and odds ratio (for categorical data) and their 95% confidence intervals will be calculated and reported.

Sensitivity analysis:

If considered useful after consultation with the review team, sensitivity analysis will be conducted to investigate the potential impact of risk of bias and participant characteristics on the effect estimates. The analysis will be restricted to different risk of bias levels to assess if study quality influences the effect estimates.

Assessment of heterogeneity and reporting bias:

Heterogeneity will be assessed using the I^2 statistic (and 95% confidence interval). Heterogeneity will be categorised as low (0-30%), moderate (30-60%), substantial (60-90%) and considerable (90-100%).[40] In accordance with Cochrane recommendations, a funnel plot will be reported to assess the presence of publication bias.

For peer review only

Analysis of subgroups or subsets:

In the presence of sufficient data, subgroup analysis will compare:

- Population characteristics (e.g. existing co-morbidities, age, gender, degree of excess weight (overweight vs. obese)).
- Intervention type (e.g. diet vs. exercise vs. diet and exercise combination; including vs. excluding psychological therapies).
- Intervention duration (e.g. 1 day, 12 weeks, 52 weeks).
- Intervention delivery format (e.g. face-to-face vs. remote; individual vs. group based).
- Comparator type (e.g. intensities of comparator – minimal/inactive/standard care).

If considered useful after consultation with the review team and in the presence of sufficient data on important covariates, meta-regression techniques will be applied to identify and/or adjust for potential sources of heterogeneity.

Narrative synthesis:

Meta-analysis will be deemed inappropriate if significant heterogeneity is present or if we are unable to pool the outcomes. If meta-analysis is not possible, narrative synthesis and 'levels of evidence' assessment will be completed. This will be provided in the text and in a table format.

A ratings system, 'levels of evidence', will be used to draw conclusions of effectiveness. This will assess confidence in cumulative evidence at an outcome level. This is based on the methods applied by a previous review paper,[41] and is modified for the synthesis of randomised controlled trials only (Supplement C). Included studies will be assessed on the level of evidence according to study quality and sample size. There are 5 possible levels of evidence ratings that can be achieved – strong, moderate, limited, inconclusive, and no evidence for effect. Consistent positive findings in at least two thirds of studies is required to achieve 'strong', 'moderate' or 'limited' levels of evidence. In stratified analysis, we will assess study's levels of evidence according to intervention, participant or study characteristics. If meta-analysis is deemed inappropriate, we will graphically summarise our findings using harvest plots of extracted data.[42]

Patient and Public Involvement (PPI)

For peer review only

A lay summary of the proposed plan for the systematic review was shared with an established PPI panel. The PPI panel gave feedback on the usefulness and relevance of the review aims and included outcomes. Upon review completion, the PPI panel will provide input on the lay summary of review findings and dissemination of findings.

ETHICS AND DISSEMINATION

Ethical approval is not required as primary data will not be collected. This systematic review will follow the PRISMA checklist. The completed systematic review will be disseminated in a peer-reviewed journal, at conferences and contribute towards the lead author's PhD thesis. The findings of the review will be of interest to participants of interventions, healthcare practitioners, policymakers, and researchers.

Acknowledgements

The authors would like to thank the University of Cambridge School of Clinical Medicine librarian Eleanor Barker for assistance in developing the search strategy. We would like to thank the patient and public involvement (PPI) representatives who contributed to the development of this research.

Author contributions

RAJ conceived the study, designed the study, developed the initial search strategy and was responsible for drafting the manuscript. ALA conceived the study, participated in study design, development of the search strategy and review drafts of the manuscript. ERL participated in study design, development of the search strategy and review drafts of the manuscript. EMFvS and SJG contributed to the design of the study and reviewed drafts of the manuscript. All authors critically reviewed the manuscript and approved the final version submitted for publication.

Funding

RAJ, ERL, ALA and SJG are supported by the Medical Research Council (MRC) (Grant MC_UU_12015/4). The University of Cambridge has received salary support in respect of SJG from the National Health Service in the East of England through the Clinical Academic Reserve. EMFvS is

For peer review only

supported by the Medical Research Council (MRC) (Grant MC_UU_12015/7). Funders had no involvement in study design, writing of the report or decision to submit the paper for publication.

Competing interests: None declared.

Patient consent: Not required.

References

1 NICE. Obesity: identification, assessment and management. Clinical guideline [CG189]. 2014.https://www.nice.org.uk/guidance/cg189/ (accessed 21 Nov 2018).

2 World Health Organisation. Obesity and overweight. 2018.http://www.who.int/news-room/fact-sheets/detail/obesity-and-overweight (accessed 21 Nov 2018).

3 Fang X, Wei J, He X, *et al.* Quantitative association between body mass index and the risk of cancer: A global Meta-analysis of prospective cohort studies. *Int J Cancer* 2018;**143**:1595–603. doi:10.1002/ijc.31553

4 Flegal K, Kit B, Orpana H, *et al.* Association of all-cause mortality with overweight and obesity using standard body mass index categories: a systematic review and meta-analysis. *JAMA* 2013;**309**:71–82. doi:10.1001/jama.2012.113905

5 Laxy M, Holle R, Döring A, *et al.* The longitudinal association between weight change and health-related quality of life: The KORA S4/F4 cohort study. *Int J Public Health* 2014;**59**:279–88. doi:10.1007/s00038-013-0506-x

6 Geiker NRW, Astrup A, Hjorth MF, *et al.* Does stress influence sleep patterns, food intake, weight gain, abdominal obesity and weight loss interventions and vice versa? *Obes Rev* 2018;**19**:81–97. doi:10.1111/obr.12603

7 Bak M, Fransen A, Janssen J, *et al.* Almost all antipsychotics result in weight gain: a meta-analysis. *PLoS One* 2014;**9**:e94112. doi:10.1371/journal.pone.0094112

8 Fruh SM. Obesity: Risk factors, complications, and strategies for sustainable long-term weight management. *J Am Assoc Nurse Pract* 2017;**29**:S3–14. doi:10.1002/2327-6924.12510

9 Fabricatore A, Wadden T, Higginbotham A, *et al.* Intentional weight loss and changes in symptoms of depression: a systematic review and meta-analysis. *Int J Obes* 2011;**35**:1363–76.

For peer review only

doi:10.1038/ijo.2011.2

10 McKibbin CL, Kitchen KA, Wykes TL, *et al.* Barriers and facilitators of a healthy lifestyle among persons with serious and persistent mental illness: Perspectives of community mental health providers. *Community Ment Health J* 2014;**50**:566–76. doi:10.1007/s10597-013-9650-2

11 Alhalel N, Schueller SM, O'Brien MJ. Association of changes in mental health with weight loss during intensive lifestyle intervention: does the timing matter? *Obes Sci Pract* 2018;**4**:153–8. doi:10.1002/osp4.157

12 Webber KH, Casey EM, Mayes L, *et al.* A comparison of a behavioral weight loss program to a stress management program: A pilot randomized controlled trial. *Nutrition* 2016;**32**:904–9. doi:10.1016/j.nut.2016.01.008

13 Fuller NR, Burns J, Sainsbury A, *et al.* Examining the association between depression and obesity during a weight management programme. *Clin Obes* 2017;**7**:354–9. doi:10.1111/cob.12208

14 Janney CA, Greenberg JM, Moin T, *et al.* Does mental health influence weight loss in adults with prediabetes? Findings from the VA Diabetes Prevention Program. *Gen Hosp Psychiatry* 2018;**53**:32–7. doi:10.1016/j.genhosppsych.2018.04.001

15 Young MD, Morgan PJ. Effect of a gender-tailored ehealth weight loss program on the depressive symptoms of overweight and obese men: pre-post study. *J Med Internet Res* 2018;**20**:1–7. doi:10.2196/mental.8920

16 Peckmezian T, Hay P. A systematic review and narrative synthesis of interventions for uncomplicated obesity: weight loss, well-being and impact on eating disorder. *J Eat Disord* 2017;**5**:212–3. doi:10.1016/j.enfcli.2017.08.004

17 Walls HL, Peeters A, Proietto J, *et al.* Public health campaigns and obesity - A critique. *BMC Public Health* 2011;**11**:1–7. doi:10.1186/1471-2458-11-136

18 Seow LSE, Chua BY, Xie H, *et al.* Correct recognition and continuum belief of mental disorders in a nursing student population. *BMC Psychiatry* 2017;**17**:1–8. doi:10.1186/s12888-017-1447-3

19 Angermeyer MC, Millier A, Rémuzat C, *et al.* Continuum beliefs and attitudes towards people with mental illness: Results from a national survey in France. *Int J Soc Psychiatry* 2015;**61**:297–303. doi:10.1177/0020764014543312

20 Schomerus G, Angermeyer MC, Baumeister SE, *et al.* An online intervention using

For peer review only

information on the mental health-mental illness continuum to reduce stigma. *Eur Psychiatry* 2016;**32**:21–7. doi:10.1016/j.eurpsy.2015.11.006

21 Pearl RL. Weight bias and stigma: public health implications and structural solutions. *Soc Issues Policy Rev* 2018;**12**:146–82. doi:10.1111/sipr.12043

22 Lazzeretti L. Assessment of psychological predictors of weight loss: How and what for? *World J Psychiatry* 2015;**5**:56. doi:10.5498/wjp.v5.i1.56

23 Rand K, Vallis M, Aston M, *et al.* 'It is not the diet; it is the mental part we need help with.' A multilevel analysis of psychological, emotional, and social well-being in obesity. *Int J Qual Stud Health Well-being* 2017;**12**. doi:10.1080/17482631.2017.1306421

24 Warkentin LM, Das D, Majumdar SR, *et al.* The effect of weight loss on health-related quality of life: Systematic review and meta-analysis of randomized trials. *Obes Rev* 2014;**15**:169–82. doi:10.1111/obr.12113

25 Lasikiewicz N, Myrissa K, Hoyland A, *et al.* Psychological benefits of weight loss following behavioural and/or dietary weight loss interventions. A systematic research review. *Appetite* 2014;**72**:123–37. doi:10.1016/j.appet.2013.09.017

26 Booth AO, Wang X, Turner AI, *et al.* Diet-induced weight loss has no effect on psychological stress in overweight and obese adults: A meta-analysis of randomized controlled trials. *Nutrients* 2018;**10**. doi:10.3390/nu10050613

27 Baillot A, Saunders S, Brunet J, *et al.* A systematic review and meta-analysis of the effect of exercise on psychosocial outcomes in adults with obesity: A call for more research. *Ment Health Phys Act* 2018;**14**:1–10. doi:10.1016/j.mhpa.2017.12.004

28 Blaine BE, Rodman J, Newman JM. Weight loss treatment and psychological well-being: A review and meta-analysis. *J Health Psychol* 2007;**12**:66–82. doi:10.1177/1359105307071741

29 Jacob A, Moullec G, Lavoie KL, *et al.* Impact of cognitive-behavioral interventions on weight loss and psychological outcomes: A meta-analysis. *Heal Psychol* 2018;**37**:417–32. doi:10.1037/hea0000576

30 Moher D, Liberati A, Tetzlaff J, *et al.* Preferred Reporting Items for Systematic Reviews and Meta-Analyses: The PRISMA Statement. *BMJ* 2009;**339**:b2535. doi:10.1136/bmj.b2535

31 Lawlor ER, Islam N, Griffin SJ, *et al.* Third-wave cognitive behaviour therapies for weight management: systematic review and network meta-analysis protocol. *BMJ Open* 2018;**8**:e023425. doi:10.1136/bmjopen-2018-023425

For peer review only

32 Loveman E, Jl C, Mead E, *et al.* Diet, physical activity and behavioural interventions for the treatment of overweight or obese adolescents aged 12 to 17 years (Review). *Cochrane* Published Online First: 2017. doi:10.1002/14651858.CD012691.www.cochranelibrary.com

33 van Dammen L, Wekker V, de Rooij SR, *et al.* A systematic review and meta-analysis of lifestyle interventions in women of reproductive age with overweight or obesity: the effects on symptoms of depression and anxiety. *Obes Rev* 2018;:1679–87. doi:10.1111/obr.12752

34 Mirza I, Pit SW. Exercise for positive mental health outcomes in adults (Protocol). *Cochrane Database Syst Rev* Published Online First: 2006. doi:10.1002/14651858.CD005615

35 The Cochrane Public Health Group. Cochrane public health group data extraction and assessment template. 2011;:1–22.

36 Schulz KF, Altman DG, Moher D. CONSORT 2010 Statement: updated guidelines for reporting parallel group randomised trials. *BMJ* 2010;**340**:c332.

37 Hoffmann TC, Glasziou PP, Boutron I, *et al.* Better reporting of interventions: template for intervention description and replication (TIDieR) checklist and guide. *BMJ* 2014;**348**:g1687. doi:10.1136/bmj.g1687

38 Higgins J, Green S, editors. *Cochrane Handbook for Systematic Reviews of Interventions Version 5.1.0 [updated March 2011]*. The Cochrane Collaboration 2011. www.handbook.cochrane.org

39 Cohen J. *Statistical power analysis for the behavioral sciences*. 2nd edn. Lawrence Erlbaum Associates 1998.

40 Ryan R, Group CC and CR. Heterogeneity and subgroup analyses in Cochrane Consumers and Communication Group reviews: Planning the analysis at protocol stage. Cochrane Consum. Commun. Rev. 2016.http://cccrg.cochrane.org (accessed 8 Jan 2019).

41 Van Sluijs EMF, McMinn AM, Griffin SJ. Effectiveness of interventions to promote physical activity in children and adolescents: Systematic review of controlled trials. *Br J Sports Med* 2008;**42**:653–7. doi:10.1136/bmj.39320.843947.BE

42 Ogilvie D, Fayter D, Petticrew M, *et al.* The harvest plot: A method for synthesising evidence about the differential effects of interventions. *BMC Med Res Methodol* 2008;**8**:1–7. doi:10.1186/1471-2288-8-8

For peer review only

Supplement A: Completed PRISMA-P Checklist

The impact of adult weight management interventions on mental health: a systematic review and meta-analysis protocol. Rebecca A. Jones, Emma R. Lawlor, Simon J. Griffin, Esther M.F. van Sluijs, Amy L. Ahern.

PRISMA-P (Preferred Reporting Items for Systematic review and Meta-Analysis Protocols) 2015 checklist: recommended items to address in a systematic review protocol*

Section and topic	Item No	Checklist item	Location in text
**ADMINISTRATIVE INFORMATION**			
Title:			
Identification	1a	Identify the report as a protocol of a systematic review	Pg. 1
Update	1b	If the protocol is for an update of a previous systematic review, identify as such	N/A
Registration	2	If registered, provide the name of the registry (such as PROSPERO) and registration number	Pg. 2 (abstract)
Authors:			
Contact	3a	Provide name, institutional affiliation, e-mail address of all protocol authors; provide physical mailing address of corresponding author	Pg. 1
Contributions	3b	Describe contributions of protocol authors and identify the guarantor of the review	Pg. 12
Amendments	4	If the protocol represents an amendment of a previously completed or published protocol, identify as such and list changes; otherwise, state plan for documenting important protocol amendments	N/A
Support:			
Sources	5a	Indicate sources of financial or other support for the review	Pg. 12/13
Sponsor	5b	Provide name for the review funder and/or sponsor	Pg. 12/13
Role of sponsor or funder	5c	Describe roles of funder(s), sponsor(s), and/or institution(s), if any, in developing the protocol	Pg. 12/13
**INTRODUCTION**			
Rationale	6	Describe the rationale for the review in the context of what is already known	Pg. 4-5
Objectives	7	Provide an explicit statement of the question(s) the review will address with reference to participants, interventions, comparators, and outcomes (PICO)	Pg. 5
**METHODS**			
Eligibility criteria	8	Specify the study characteristics (such as PICO, study design, setting, time frame) and report characteristics (such as years considered, language, publication status) to be used as criteria for eligibility for the review	Pg. 6-7

For peer review only

Information sources	9	Describe all intended information sources (such as electronic databases, contact with study authors, trial registers or other grey literature sources) with planned dates of coverage	Pg. 7-8
Search strategy	10	Present draft of search strategy to be used for at least one electronic database, including planned limits, such that it could be repeated	Supp. B
Study records:			
Data management	11a	Describe the mechanism(s) that will be used to manage records and data throughout the review	Pg. 8
Selection process	11b	State the process that will be used for selecting studies (such as two independent reviewers) through each phase of the review (that is, screening, eligibility and inclusion in meta-analysis)	Pg. 8
Data collection process	11c	Describe planned method of extracting data from reports (such as piloting forms, done independently, in duplicate), any processes for obtaining and confirming data from investigators	Pg. 8
Data items	12	List and define all variables for which data will be sought (such as PICO items, funding sources), any pre-planned data assumptions and simplifications	Pg. 9
Outcomes and prioritization	13	List and define all outcomes for which data will be sought, including prioritization of main and additional outcomes, with rationale	Pg. 9
Risk of bias in individual studies	14	Describe anticipated methods for assessing risk of bias of individual studies, including whether this will be done at the outcome or study level, or both; state how this information will be used in data synthesis	Pg. 9-10
Data synthesis	15a	Describe criteria under which study data will be quantitatively synthesised	Pg. 10-11
	15b	If data are appropriate for quantitative synthesis, describe planned summary measures, methods of handling data and methods of combining data from studies, including any planned exploration of consistency (such as I^2, Kendall's τ)	Pg. 10-11
	15c	Describe any proposed additional analyses (such as sensitivity or subgroup analyses, meta-regression)	Pg. 10-11
	15d	If quantitative synthesis is not appropriate, describe the type of summary planned	Pg. 11
Meta-bias(es)	16	Specify any planned assessment of meta-bias(es) (such as publication bias across studies, selective reporting within studies)	Pg. 11
Confidence in cumulative evidence	17	Describe how the strength of the body of evidence will be assessed (such as GRADE)	Pg. 11

*** It is strongly recommended that this checklist be read in conjunction with the PRISMA-P Explanation and Elaboration (cite when available) for important clarification on the items. Amendments to a review protocol should be tracked and dated. The copyright for PRISMA-P (including checklist) is held by the PRISMA-P Group and is distributed under a Creative Commons Attribution Licence 4.0.**

From: Shamseer L, Moher D, Clarke M, Ghersi D, Liberati A, Petticrew M, Shekelle P, Stewart L, PRISMA-P Group. Preferred reporting items for systematic review and meta-analysis protocols (PRISMA-P) 2015: elaboration and explanation. BMJ. 2015 Jan 2;349(jan02 1):g7647.

For peer review only

Supplement B: Search Strategy (Medline)

(1) Participants	1.	exp Overweight/ or exp Obesity/ or (adipos* or obes* or over?weight).tw
(2) Weight management intervention	2.	exp Body Weight/ or exp Life Style/ or exp Physical Activity/ or exp Obesity Management/ or exp Diet Therapy/ or exp Exercise/ or exp Diet/ or exp Behavior Therapy/ or exp Health Education/ or ((weight adj3 (body or chang* or los* or maint* or manage* or control* or reduc*)) or (body?mass?index or bmi) or (body adj3 mass) or life?style or (diet* or nutrition*) or (physic* adj3 (activ* or fit*)) or exercis* or (obes* adj3 (intervention or program* or camp* or treat*)) or (behavio?r* or psych*)).tw
(3) Mental health outcomes	3.	exp Behavioral Symptoms/ or exp Emotions/ or exp Mental Disorders/ or exp Adaptation, Psychological/ or exp Mental Health/ or exp Quality of Life/ or exp Self Concept/ OR (depress* or anxiet* or well?being or (quality?of?life or qol or health?status) or (affect* or mood*) or (health?related?quality?of?life or hrqol) or emotion* or (mental adj3 (health or well?being)) or (psych* adj3 (well?being or health)) or self?esteem or self?image or body?image or stress* or (emot* adj3 eating) or binge?eating).tw
(4) Study design	4.	exp Randomized Controlled Trials as Topic/ or randomized controlled trial.pt or controlled clinical trial.mp or randomi?ed.mp or randomly.mp or trial.mp
	5.	1 AND 2 AND 3 AND 4

Limit (5) to English-language results.

For peer review only

Supplement C: Levels of evidence assessment

Modified flow chart of the decision-making process for levels of evidence - based on study quality and study size. Consistent positive results (66.6% of relevant studies reporting significant positive results) are needed to achieve strong, moderate or limited levels of evidence.

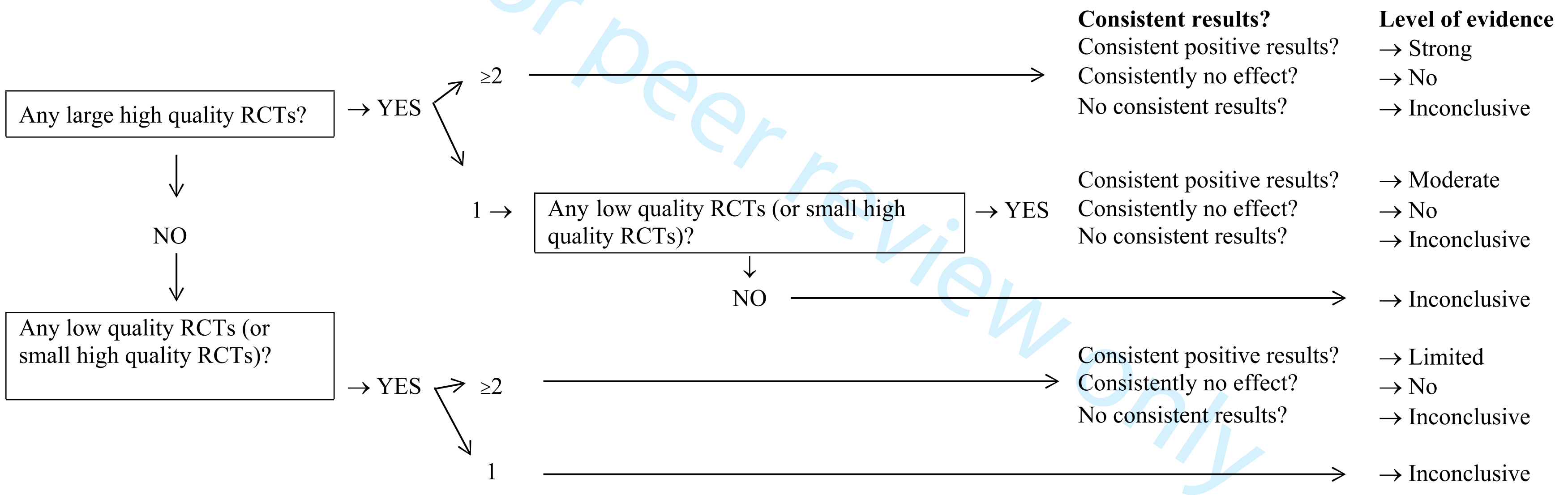

NOTE: studies including ≤ 250 participants or not providing sample size justifying a smaller sample size are considered 'small', studies including >250 participants are considered 'large'.

For peer review only

BMJ Open

The impact of adult weight management interventions on mental health: a systematic review and meta-analysis protocol.

Journal:	*BMJ Open*
Manuscript ID	bmjopen-2019-031857.R1
Article Type:	Protocol
Date Submitted by the Author:	22-Nov-2019
Complete List of Authors:	Jones, Rebecca; University of Cambridge, MRC Epidemiology Unit<br>Lawlor, Emma; MRC Epidemiology Unit, University of Cambridge<br>Griffin, Simon; The Primary Care Unit, Institute of Public Health; University of Cambridge, MRC Epidemiology Unit<br>van Sluijs, Esther; University of Cambridge, MRC Epidemiology Unit; University of Cambridge, UKCRC Centre for Diet and Activity Research (CEDAR)<br>Ahern, Amy; MRC Epidemiology Unit, University of Cambridge
**Primary Subject Heading**:	Public health
Secondary Subject Heading:	Mental health, Public health, Epidemiology
Keywords:	PUBLIC HEALTH, MENTAL HEALTH, EPIDEMIOLOGY

SCHOLARONE™
Manuscripts

BMJ

I, the Submitting Author has the right to grant and does grant on behalf of all authors of the Work (as defined in the below author licence), an exclusive licence and/or a non-exclusive licence for contributions from authors who are: i) UK Crown employees; ii) where BMJ has agreed a CC-BY licence shall apply, and/or iii) in accordance with the terms applicable for US Federal Government officers or employees acting as part of their official duties; on a worldwide, perpetual, irrevocable, royalty-free basis to BMJ Publishing Group Ltd ("BMJ") its licensees and where the relevant Journal is co-owned by BMJ to the co-owners of the Journal, to publish the Work in this journal and any other BMJ products and to exploit all rights, as set out in our licence.

The Submitting Author accepts and understands that any supply made under these terms is made by BMJ to the Submitting Author unless you are acting as an employee on behalf of your employer or a postgraduate student of an affiliated institution which is paying any applicable article publishing charge ("APC") for Open Access articles. Where the Submitting Author wishes to make the Work available on an Open Access basis (and intends to pay the relevant APC), the terms of reuse of such Open Access shall be governed by a Creative Commons licence – details of these licences and which Creative Commons licence will apply to this Work are set out in our licence referred to above.

Other than as permitted in any relevant BMJ Author's Self Archiving Policies, I confirm this Work has not been accepted for publication elsewhere, is not being considered for publication elsewhere and does not duplicate material already published. I confirm all authors consent to publication of this Work and authorise the granting of this licence.

For peer review only

The impact of adult weight management interventions on mental health: a systematic review and meta-analysis protocol.

Rebecca A. Jones[1], Emma R. Lawlor[1], Simon J. Griffin[1,2], Esther M.F. van Sluijs[1,3], Amy L. Ahern[1]

Corresponding author:

Rebecca A. Jones - rj397@cam.ac.uk / MRC Epidemiology Unit, University of Cambridge, Cambridge, UK.

Author affiliations:

[1] MRC Epidemiology Unit, University of Cambridge, Cambridge, UK.

[2] Primary Care Unit, Institute of Public Health, University of Cambridge, Cambridge, UK.

[3] UKCRC Centre for Diet and Activity Research (CEDAR), University of Cambridge, Cambridge, UK.

For peer review only

Abstract word count: 283/300 words

Full text word count: 3412/4000 words

ABSTRACT

Introduction: The effects of interventions targeting weight loss on physical health are well-described, yet the evidence for mental health is less clear. It is essential to better understand the impact of weight management interventions on mental health to optimise care and minimise risk of harm. We will assess the effect of behavioural weight management interventions on mental health in adults with overweight and obesity.

Methods and analysis: The systematic review will follow the Preferred Reporting Items for Systematic Reviews and Meta-analyses guidance. We will include behavioural weight management interventions with a diet and/or physical activity component focusing on weight loss for adults with a body mass index (BMI) ≥25 kg/m^2. Randomised controlled trials (RCT) and cluster RCTs will be the only eligible study designs. Outcomes of interest will be related to mental health. The following databases were searched from inception to 07/05/2019: MEDLINE, Embase, Cochrane database (CENTRAL), PsycINFO, ASSIA, AMED and CINAHL. The search strategy was based on four concepts: (1) Overweight/obese adults defined as ≥18 years, (2) Weight management interventions, (3) Mental health outcomes, and (4) Study design. The search was restricted to English-language published papers, with no other restrictions applied. Two stage screening for eligibility will be completed by two independent reviewers, with two independent reviewers completing data extraction and risk of bias assessment. Data permitting, a random-effects meta-analysis of outcomes, sub-group analyses and meta-regression will be conducted. If not appropriate, narrative synthesis and 'levels of evidence' assessment will be completed.

Ethics and dissemination: Ethical approval is not required as primary data will not be collected. The completed systematic review will be disseminated in a peer-reviewed journal, at conferences and contribute towards the lead author's PhD thesis.

PROSPERO registration number: CRD42019131659

For peer review only - http://bmjopen.bmj.com/site/about/guidelines.xhtml

ARTICLE SUMMARY

Strengths and limitations of this study

- The systematic review and meta-analysis will include only randomised controlled trials, offering the highest level of evidence.
- A broad array of mental health outcomes, including mood, stress and depression, will be included in the review.
- A comprehensive search strategy will be used in a large number of databases to maximise the identification of all eligible studies.
- Data permitting, sub-group analysis will be conducted to identify intervention or participant characteristics associated with increased effectiveness.
- High heterogeneity is anticipated across studies which may increase the difficulties in interpreting a meta-analysis.

For peer review only

INTRODUCTION

Rationale

Overweight and obesity is strongly associated with reduced physical health, including a greater risk of cardiovascular disease, type 2 diabetes, stroke, and some cancers (including endometrial, oesophageal and kidney cancer).[1–3] Consequently, individuals with overweight and obesity experience greater all-cause mortality and reduced health-related quality of life.[4,5] Research reports a bidirectional association between obesity and mental health, with those with overweight and obese more likely to have poor mental health and those with mental ill-health at greater risk of weight gain, and consequently, obesity.[6–10] Many have researchers reported improvements in mental health outcomes with weight loss,[11–15] however there has been concern expressed that weight management interventions advocating dietary restriction may contribute to disordered eating and worsen mental health.[16,17] It is essential to better understand the impact of weight management interventions on mental health to optimise care and minimise risk of harm.

Research investigating the relationship between obesity and mental health is increasingly considering mental health as a symptom continuum. The symptom continuum appreciates that individuals can experience one or more symptoms of mental illness without meeting diagnostic criteria for mental illness.[18,19] Considering mental health as a continuum is associated with reduced stigma and improved attitudes towards mental health, highlighting the benefits of broadening the definition of mental health.[19,20] This review will embrace a continuum-based definition of mental health allowing the investigation of a broader range of outcomes from stress, self-efficacy and affect, to symptoms of clinically diagnosed disorders such as depression and anxiety.

While there is clear evidence that weight loss interventions improve physical health, the evidence that they enhance mental health is less clear. Some studies suggest that a focus on weight control can increase stigma and exacerbate symptoms of psychological distress,[21] particularly if goals are not met or if other aspects of life do not change with weight loss.[22] Qualitative research has suggested that there is inadequate support for mental health in obesity management interventions,[23] and a systematic review published in 2014 concluded that weight loss may be associated with improved physical health, but not mental health.[24] Conversely, Fabricatore et al.'s review found statistically significant reductions in depressive symptoms with intentional weight loss trials, although it reported no relationship between weight change and depression,[9] and Lasikiewicz and colleagues' review reported weight management interventions to be associated with improvements in multiple mental health outcomes including self-esteem, body image, quality of life and depressive symptoms.[25]

Previous reviews highlight the breadth of mental health outcomes that could be affected by participation in weight management programmes. However, the majority of reviews focus on a

For peer review only - http://bmjopen.bmj.com/site/about/guidelines.xhtml

limited range of outcomes,[9,16,24–28] and the direction of effects is inconsistent across different outcomes and reviews. It is important to generate a comprehensive understanding of the impact of weight management programmes on mental health as the benefits of improvements in one domain may be undermined by negative impacts on other domains. Previous reviews have also excluded participants with any concurrent disease or clinical psychopathology to constrain the search or to exclude illnesses associated with unintentional weight changes.[16,29] However it is uncommon for an individual with overweight or obesity to be without any concurrent disease or clinical psychopathology due to the greatly increased risk of a wide range of comorbidities,[8] therefore exclusion of these participants limits the representativeness of findings. It is considered beneficial to include participants with comorbid conditions where possible to maximise the generalisability of review findings.

To our knowledge, there is no up-to-date, comprehensive review investigating the effect of weight management interventions on a broad range of mental health outcomes in a representative sample of adults with overweight and obesity. Furthermore, no review has investigated the intervention components most supportive of mental health improvements. Understanding whether intervention components, such as psychological aspects, can attenuate the possible adverse effects to mental health is important for the development of future interventions. This systematic review will apply sub-group analyses and meta-regression techniques to explore the differential effects of intervention or participant characteristics on mental health.

The conflicting findings of previous research and the absence of an up-to-date evaluation of the impact of weight loss interventions on mental health make it difficult to draw clear, reliable conclusions. A comprehensive updated review should increase understanding of the impact of weight management interventions on mental health. The most effective combination of intervention components should be investigated to facilitate improved decision-making in intervention development, aiding the creation of an effective and supportive 'whole-person' intervention.

Objectives

To assess the effectiveness of behavioural weight management interventions compared to minimal, inactive or 'standard care' control groups on mental health in adults with overweight and obesity.

Primary objective*:* (1) Quantify the effect of behavioural weight management interventions on mental health in adults with overweight and obesity.

Secondary objective: (2) Quantify if particular intervention or participant characteristics influence the effect of interventions on mental health.

For peer review only - http://bmjopen.bmj.com/site/about/guidelines.xhtml

METHODS AND ANALYSIS

This systematic review protocol adhered to the PRISMA-P reporting guidelines for systematic review and meta-analysis protocols (Supplement A).[30]

Eligibility criteria

Studies will be selected according to the criteria outlined below:

Study designs:

Original peer-reviewed primary research articles reporting randomised controlled trials (RCTs) or cluster RCTs will be included. No restrictions will be placed on year of publication.

Participants:

Participants will be included if they are community-dwelling adults (≥ 18 years old with no upper age limit applied) with overweight or obesity (body mass index (BMI) ≥ 25 kg/m^2) at baseline. Studies that include participants both under and over the age of 18 years will only be included if the data for participants 18 years and older is reported separately. Participants must be seeking intentional weight loss through a behavioural programme. No restrictions will be made on participant demographics. To increase the generalisability of the findings to the general population with overweight and obesity, we will include studies that include people with comorbidities but we will exclude papers that focus exclusively on populations with a physical or mental comorbidity (e.g. all participants have cancer), or pregnant women.

Interventions:

Studies will be included if they evaluated a behavioural weight management intervention that aims to achieve weight loss through changes in diet and/or physical activity. No restriction will be placed on intervention delivery duration, delivery format or on who delivers the intervention. Any study with multiple intervention arms will be included if at least one arm meets the inclusion criteria and separate results are presented for this arm. Interventions aiming to treat eating disorders or involving surgical and/or pharmacological intervention will be excluded.

Comparators:

Studies with a minimal/inactive/standard care control group will be included.

Outcomes:

Included studies are required to have measures of one or more of the following outcomes: Quality of life; Mood/Affect; Stress; Self-esteem; Body image; Emotional eating; Binge eating; Depression;

For peer review only - http://bmjopen.bmj.com/site/about/guidelines.xhtml

For peer review only

Anxiety. These a-priori defined outcomes were chosen as they were deemed to be the most relevant and frequently used in previous relevant literature.

Timing:

Defined outcomes must be measured and reported at pre-intervention and at minimum one follow-up point to be eligible for inclusion. The follow-up measurements closest to the time of intervention completion will be extracted for analysis to focus on the immediate intervention effects.

Settings:

Only studies involving participants living in community-based settings will be included.

Language:

Studies published in English language will be included. Non-English language publications will be excluded.

Information sources and search strategy

The following databases were searched from inception to 7th May 2019:

- AMED
- ASSIA
- CINAHL
- Cochrane database (CENTRAL)
- Embase
- MEDLINE
- PsycINFO

Detailed search strategies for each electronic database were developed by RAJ, who has previous experience of conducting systematic reviews, with input from ERL, ALA and a medical librarian. The search strategy contains relevant key words and headings based on previous review articles [25,31–34] and is based on the concepts: (1) Overweight/obese adults AND (2) Weight management interventions AND (3) Mental health outcomes AND (4) Study designs. Terms were adapted from the MEDLINE search accordingly for each database (Supplement B). The search was restricted to English-language papers, with no other restrictions applied. The search strategy was validated through consultation with the systematic review team.

For peer review only - http://bmjopen.bmj.com/site/about/guidelines.xhtml

Other resource searches:

To augment the results of the database search, the reference lists of included studies and previous relevant reviews will be searched.[9,16,24–27,29]

Study records

Data management and selection process:

The search results were imported into Covidence systematic review software (Veritas Health Innovation, Melbourne, Australia), and duplications removed. Two researchers initially pilot screened an identical 500 articles to ensure consistency. Any discrepancies in the interpretation of the eligibility criteria were discussed between investigators, with a third reviewer assisting where necessary. Upon completion of pilot screening, the remaining title and abstracts will be independently screened for inclusion by two authors. The full-text of articles identified as potentially relevant will be obtained and dually screened according to the eligibility criteria to ascertain the studies to be included in the review. Eligibility will be discussed for consensus between the two investigators, with a third investigator resolving discrepancies when required. Where necessary, we will seek additional information from study authors to resolve any questions about eligibility. Reasons for exclusion of articles at the full-text screening stage will be recorded. Reviewers will not be blinded to authors, institution or journal when screening articles.

Where studies are reported in more than one publication, all articles will be included and combined to make best use of the data. A Preferred Reporting Items for Systematic Reviews and Meta-Analyses (PRISMA) flow chart will be reported to show the process of study selection.[30]

Data collection process:

Studies meeting the inclusion criteria will have pertinent data extracted using a data extraction form. The data extraction form will be based on the Cochrane data extraction form (2011),[35] the Consolidated Standards of Reporting Trials (CONSORT) statement (2010),[36] and the Cochrane Template for Intervention Description and Replication (TIDieR) [37] to ensure breadth and detail will be captured. The data extraction form will be pilot tested by two investigators on three studies to identify missing or superfluous data items. Independent data extraction will be completed by one investigator with full checking by a minimum of further one investigator. Discrepancies will be resolved through discussion, with use of a third investigator where necessary.

For peer review only - http://bmjopen.bmj.com/site/about/guidelines.xhtml

Data items

Data to be extracted will include:

- General information – e.g. study authors, publication year, country, funding source.
- Study details – e.g. study aim, study design, randomisation method, blinding and allocation concealment.
- Participant information – e.g. demographics, recruitment methods, sample size, co-morbidities.
- Attrition/adherence – e.g. total number of participants at baseline and follow up measurements, differential attrition, attendance, study withdrawal, loss to follow-up.
- Intervention information – e.g. setting, content, intervention duration and frequency, profession delivering the intervention, method of delivery, group or individual delivery.
- Comparator information – e.g. setting, content, intervention duration and frequency, profession delivering the intervention, method of delivery, group or individual delivery.
- Outcomes – e.g. mental health outcome(s) studied, whether self-reported or objectively measured, duration of follow-up, statistical analysis, intervention effect sizes.

If a study has multiple arms, data from any arm meeting the inclusion criteria will be extracted where possible. Study authors will be contacted if there are uncertainties regarding the study or missing data.

Outcomes and prioritisation

For all outcomes, prioritisation will be given to units reported as raw data at baseline and post-intervention over data presented as 'mean change' or equivalent. Where possible, data items will be extracted at both study and group level to permit analysis of overall and stratified data (e.g. extracting stratified data to analyse moderation by sex). Study authors will be contacted to request any data required that is not available.

Risk of bias in individual studies

Risk of bias will be independently appraised by a minimum of two review authors. Discrepancies will be discussed between authors for a consensus and a third investigator will be consulted where required.

For peer review only - http://bmjopen.bmj.com/site/about/guidelines.xhtml

The Cochrane 'Risk of bias' tool (RoB) will be used to assess the risk of bias in the included studies.[38] The tool assesses the following study features as 'low risk', 'high risk' or 'unclear': (1) Random sequence generation, (2) Allocation concealment, (3) Blinding of participants and personnel, (4) Blinding of outcome assessment, (5) Incomplete outcome data, and (6) Selective reporting.

Other potential sources of bias not covered by the tool will be noted by review authors. Review authors will not be blinded to the included study's information (author names, journal of publication, affiliated institute). A risk of bias graph and summary table will be presented.

Data synthesis

When the data permits, outcome data will be synthesised using a random-effects meta-analysis (Review Manager v5.3, Cochrane Collaboration) due to the predicted diverse range of population and intervention types. Meta-analysis will be conducted on the outcome measures reported closest to the time of intervention completion, regardless of intervention duration, to focus analysis on the immediate intervention effects.

As it is likely a range of outcome measures will be identified, standardised mean difference (SMD) will be calculated. SMD will be categorised using thresholds as small (0.2), medium (0.5) and large (0.8).[39] Where possible, mean differences (for continuous data) and odds ratio (for categorical data) and their 95% confidence intervals will be calculated and reported.

Sensitivity analysis:

If considered useful after consultation with the review team, sensitivity analysis will be conducted to investigate the potential impact of risk of bias and participant characteristics on the effect estimates. The analysis will be restricted to different risk of bias levels to assess if study quality influences the effect estimates.

Assessment of heterogeneity and reporting bias:

Heterogeneity will be assessed using the I^2 statistic (and 95% confidence interval). Heterogeneity will be categorised as low (0-30%), moderate (30-60%), substantial (60-90%) and considerable (90-100%).[40] In accordance with Cochrane recommendations, a funnel plot will be reported to assess the presence of publication bias.

For peer review only - http://bmjopen.bmj.com/site/about/guidelines.xhtml

Analysis of subgroups or subsets:

In the presence of sufficient data, subgroup analysis will compare:

- Population characteristics (e.g. existing co-morbidities, age, gender, degree of excess weight (overweight vs. obese)).
- Intervention type (e.g. diet vs. exercise vs. diet and exercise combination; including vs. excluding psychological therapies).
- Intervention duration (e.g. 1 day, 12 weeks, 52 weeks).
- Intervention delivery format (e.g. face-to-face vs. remote; individual vs. group based).
- Comparator type (e.g. intensities of comparator – minimal/inactive/standard care).

If considered useful after consultation with the review team and in the presence of sufficient data on important covariates, meta-regression techniques will be applied to identify and/or adjust for potential sources of heterogeneity.

Narrative synthesis:

Meta-analysis will be deemed inappropriate if significant heterogeneity is present or if we are unable to pool the outcomes. If meta-analysis is not possible, narrative synthesis and 'levels of evidence' assessment will be completed. This will be provided in the text and in a table format.

A ratings system, 'levels of evidence', will be used to draw conclusions of effectiveness. This will assess confidence in cumulative evidence at an outcome level. This is based on the methods applied by a previous review paper,[41] and is modified for the synthesis of randomised controlled trials only (Supplement C). Included studies will be assessed on the level of evidence according to study quality and sample size. There are 5 possible levels of evidence ratings that can be achieved – strong, moderate, limited, inconclusive, and no evidence for effect. Consistent positive findings in at least two thirds of studies is required to achieve 'strong', 'moderate' or 'limited' levels of evidence. In stratified analysis, we will assess study's levels of evidence according to intervention, participant or study characteristics. If meta-analysis is deemed inappropriate, we will graphically summarise our findings using harvest plots of extracted data.[42]

Patient and Public Involvement (PPI)

For peer review only - http://bmjopen.bmj.com/site/about/guidelines.xhtml

A lay summary of the proposed plan for the systematic review was shared with an established PPI panel. The PPI panel gave feedback on the usefulness and relevance of the review aims and included outcomes. Upon review completion, the PPI panel will provide input on the lay summary of review findings and dissemination of findings.

ETHICS AND DISSEMINATION

Ethical approval is not required as primary data will not be collected. This systematic review will follow the PRISMA checklist. The completed systematic review will be disseminated in a peer-reviewed journal, at conferences and contribute towards the lead author's PhD thesis. The findings of the review will be of interest to participants of interventions, healthcare practitioners, policymakers, and researchers.

Acknowledgements

The authors would like to thank the University of Cambridge School of Clinical Medicine librarian Eleanor Barker for assistance in developing the search strategy. We would like to thank the patient and public involvement (PPI) representatives who contributed to the development of this research.

Author contributions

RAJ conceived the study, designed the study, developed the initial search strategy and was responsible for drafting the manuscript. ALA conceived the study, participated in study design, development of the search strategy and review drafts of the manuscript. ERL participated in study design, development of the search strategy and review drafts of the manuscript. EMFvS and SJG contributed to the design of the study and reviewed drafts of the manuscript. All authors critically reviewed the manuscript and approved the final version submitted for publication.

Funding

RAJ, ERL, ALA and SJG are supported by the Medical Research Council (MRC) (Grant MC_UU_12015/4). The University of Cambridge has received salary support in respect of SJG from the National Health Service in the East of England through the Clinical Academic Reserve. EMFvS is

For peer review only - http://bmjopen.bmj.com/site/about/guidelines.xhtml

supported by the Medical Research Council (MRC) (Grant MC_UU_12015/7). Funders had no involvement in study design, writing of the report or decision to submit the paper for publication.

Competing interests: None declared.

Patient consent: Not required.

References

1 NICE. Obesity: identification, assessment and management. Clinical guideline [CG189]. 2014.https://www.nice.org.uk/guidance/cg189/ (accessed 21 Nov 2018).

2 World Health Organisation. Obesity and overweight. 2018.http://www.who.int/news-room/fact-sheets/detail/obesity-and-overweight (accessed 21 Nov 2018).

3 Fang X, Wei J, He X, *et al.* Quantitative association between body mass index and the risk of cancer: A global Meta-analysis of prospective cohort studies. *Int J Cancer* 2018;**143**:1595–603. doi:10.1002/ijc.31553

4 Flegal K, Kit B, Orpana H, *et al.* Association of all-cause mortality with overweight and obesity using standard body mass index categories: a systematic review and meta-analysis. *JAMA* 2013;**309**:71–82. doi:10.1001/jama.2012.113905

5 Laxy M, Holle R, Döring A, *et al.* The longitudinal association between weight change and health-related quality of life: The KORA S4/F4 cohort study. *Int J Public Health* 2014;**59**:279–88. doi:10.1007/s00038-013-0506-x

6 Geiker NRW, Astrup A, Hjorth MF, *et al.* Does stress influence sleep patterns, food intake, weight gain, abdominal obesity and weight loss interventions and vice versa? *Obes Rev* 2018;**19**:81–97. doi:10.1111/obr.12603

7 Bak M, Fransen A, Janssen J, *et al.* Almost all antipsychotics result in weight gain: a meta-analysis. *PLoS One* 2014;**9**:e94112. doi:10.1371/journal.pone.0094112

8 Fruh SM. Obesity: Risk factors, complications, and strategies for sustainable long-term weight management. *J Am Assoc Nurse Pract* 2017;**29**:S3–14. doi:10.1002/2327-6924.12510

9 Fabricatore A, Wadden T, Higginbotham A, *et al.* Intentional weight loss and changes in symptoms of depression: a systematic review and meta-analysis. *Int J Obes* 2011;**35**:1363–76.

For peer review only - http://bmjopen.bmj.com/site/about/guidelines.xhtml

doi:10.1038/ijo.2011.2

10 McKibbin CL, Kitchen KA, Wykes TL, *et al.* Barriers and facilitators of a healthy lifestyle among persons with serious and persistent mental illness: Perspectives of community mental health providers. *Community Ment Health J* 2014;**50**:566–76. doi:10.1007/s10597-013-9650-2

11 Alhalel N, Schueller SM, O'Brien MJ. Association of changes in mental health with weight loss during intensive lifestyle intervention: does the timing matter? *Obes Sci Pract* 2018;**4**:153–8. doi:10.1002/osp4.157

12 Webber KH, Casey EM, Mayes L, *et al.* A comparison of a behavioral weight loss program to a stress management program: A pilot randomized controlled trial. *Nutrition* 2016;**32**:904–9. doi:10.1016/j.nut.2016.01.008

13 Fuller NR, Burns J, Sainsbury A, *et al.* Examining the association between depression and obesity during a weight management programme. *Clin Obes* 2017;**7**:354–9. doi:10.1111/cob.12208

14 Janney CA, Greenberg JM, Moin T, *et al.* Does mental health influence weight loss in adults with prediabetes? Findings from the VA Diabetes Prevention Program. *Gen Hosp Psychiatry* 2018;**53**:32–7. doi:10.1016/j.genhosppsych.2018.04.001

15 Young MD, Morgan PJ. Effect of a gender-tailored ehealth weight loss program on the depressive symptoms of overweight and obese men: pre-post study. *J Med Internet Res* 2018;**20**:1–7. doi:10.2196/mental.8920

16 Peckmezian T, Hay P. A systematic review and narrative synthesis of interventions for uncomplicated obesity: weight loss, well-being and impact on eating disorder. *J Eat Disord* 2017;**5**:212–3. doi:10.1016/j.enfcli.2017.08.004

17 Walls HL, Peeters A, Proietto J, *et al.* Public health campaigns and obesity - A critique. *BMC Public Health* 2011;**11**:1–7. doi:10.1186/1471-2458-11-136

18 Seow LSE, Chua BY, Xie H, *et al.* Correct recognition and continuum belief of mental disorders in a nursing student population. *BMC Psychiatry* 2017;**17**:1–8. doi:10.1186/s12888-017-1447-3

19 Angermeyer MC, Millier A, Rémuzat C, *et al.* Continuum beliefs and attitudes towards people with mental illness: Results from a national survey in France. *Int J Soc Psychiatry* 2015;**61**:297–303. doi:10.1177/0020764014543312

20 Schomerus G, Angermeyer MC, Baumeister SE, *et al.* An online intervention using

For peer review only - http://bmjopen.bmj.com/site/about/guidelines.xhtml

information on the mental health-mental illness continuum to reduce stigma. *Eur Psychiatry* 2016;**32**:21–7. doi:10.1016/j.eurpsy.2015.11.006

21 Pearl RL. Weight bias and stigma: public health implications and structural solutions. *Soc Issues Policy Rev* 2018;**12**:146–82. doi:10.1111/sipr.12043

22 Lazzeretti L. Assessment of psychological predictors of weight loss: How and what for? *World J Psychiatry* 2015;**5**:56. doi:10.5498/wjp.v5.i1.56

23 Rand K, Vallis M, Aston M, *et al.* 'It is not the diet; it is the mental part we need help with.' A multilevel analysis of psychological, emotional, and social well-being in obesity. *Int J Qual Stud Health Well-being* 2017;**12**. doi:10.1080/17482631.2017.1306421

24 Warkentin LM, Das D, Majumdar SR, *et al.* The effect of weight loss on health-related quality of life: Systematic review and meta-analysis of randomized trials. *Obes Rev* 2014;**15**:169–82. doi:10.1111/obr.12113

25 Lasikiewicz N, Myrissa K, Hoyland A, *et al.* Psychological benefits of weight loss following behavioural and/or dietary weight loss interventions. A systematic research review. *Appetite* 2014;**72**:123–37. doi:10.1016/j.appet.2013.09.017

26 Booth AO, Wang X, Turner AI, *et al.* Diet-induced weight loss has no effect on psychological stress in overweight and obese adults: A meta-analysis of randomized controlled trials. *Nutrients* 2018;**10**. doi:10.3390/nu10050613

27 Baillot A, Saunders S, Brunet J, *et al.* A systematic review and meta-analysis of the effect of exercise on psychosocial outcomes in adults with obesity: A call for more research. *Ment Health Phys Act* 2018;**14**:1–10. doi:10.1016/j.mhpa.2017.12.004

28 Blaine BE, Rodman J, Newman JM. Weight loss treatment and psychological well-being: A review and meta-analysis. *J Health Psychol* 2007;**12**:66–82. doi:10.1177/1359105307071741

29 Jacob A, Moullec G, Lavoie KL, *et al.* Impact of cognitive-behavioral interventions on weight loss and psychological outcomes: A meta-analysis. *Heal Psychol* 2018;**37**:417–32. doi:10.1037/hea0000576

30 Moher D, Liberati A, Tetzlaff J, *et al.* Preferred Reporting Items for Systematic Reviews and Meta-Analyses: The PRISMA Statement. *BMJ* 2009;**339**:b2535. doi:10.1136/bmj.b2535

31 Lawlor ER, Islam N, Griffin SJ, *et al.* Third-wave cognitive behaviour therapies for weight management: systematic review and network meta-analysis protocol. *BMJ Open* 2018;**8**:e023425. doi:10.1136/bmjopen-2018-023425

For peer review only - http://bmjopen.bmj.com/site/about/guidelines.xhtml

32 Loveman E, Jl C, Mead E, *et al.* Diet, physical activity and behavioural interventions for the treatment of overweight or obese adolescents aged 12 to 17 years (Review). *Cochrane* Published Online First: 2017. doi:10.1002/14651858.CD012691.www.cochranelibrary.com

33 van Dammen L, Wekker V, de Rooij SR, *et al.* A systematic review and meta-analysis of lifestyle interventions in women of reproductive age with overweight or obesity: the effects on symptoms of depression and anxiety. *Obes Rev* 2018;:1679–87. doi:10.1111/obr.12752

34 Mirza I, Pit SW. Exercise for positive mental health outcomes in adults (Protocol). *Cochrane Database Syst Rev* Published Online First: 2006. doi:10.1002/14651858.CD005615

35 The Cochrane Public Health Group. Cochrane public health group data extraction and assessment template. 2011;:1–22.

36 Schulz KF, Altman DG, Moher D. CONSORT 2010 Statement: updated guidelines for reporting parallel group randomised trials. *BMJ* 2010;**340**:c332.

37 Hoffmann TC, Glasziou PP, Boutron I, *et al.* Better reporting of interventions: template for intervention description and replication (TIDieR) checklist and guide. *BMJ* 2014;**348**:g1687. doi:10.1136/bmj.g1687

38 Higgins J, Green S, editors. *Cochrane Handbook for Systematic Reviews of Interventions Version 5.1.0 [updated March 2011]*. The Cochrane Collaboration 2011. www.handbook.cochrane.org

39 Cohen J. *Statistical power analysis for the behavioral sciences*. 2nd edn. Lawrence Erlbaum Associates 1998.

40 Ryan R, Group CC and CR. Heterogeneity and subgroup analyses in Cochrane Consumers and Communication Group reviews: Planning the analysis at protocol stage. Cochrane Consum. Commun. Rev. 2016.http://cccrg.cochrane.org (accessed 8 Jan 2019).

41 Van Sluijs EMF, McMinn AM, Griffin SJ. Effectiveness of interventions to promote physical activity in children and adolescents: Systematic review of controlled trials. *Br J Sports Med* 2008;**42**:653–7. doi:10.1136/bmj.39320.843947.BE

42 Ogilvie D, Fayter D, Petticrew M, *et al.* The harvest plot: A method for synthesising evidence about the differential effects of interventions. *BMC Med Res Methodol* 2008;**8**:1–7. doi:10.1186/1471-2288-8-8

For peer review only

For peer review only - http://bmjopen.bmj.com/site/about/guidelines.xhtml

Supplement A: Completed PRISMA-P Checklist

The impact of adult weight management interventions on mental health: a systematic review and meta-analysis protocol. Rebecca A. Jones, Emma R. Lawlor, Simon J. Griffin, Esther M.F. van Sluijs, Amy L. Ahern.

PRISMA-P (Preferred Reporting Items for Systematic review and Meta-Analysis Protocols) 2015 checklist: recommended items to address in a systematic review protocol*

Section and topic	Item No	Checklist item	Location in text
**ADMINISTRATIVE INFORMATION**			
Title:			
Identification	1a	Identify the report as a protocol of a systematic review	Pg. 1
Update	1b	If the protocol is for an update of a previous systematic review, identify as such	N/A
Registration	2	If registered, provide the name of the registry (such as PROSPERO) and registration number	Pg. 2 (abstract)
Authors:			
Contact	3a	Provide name, institutional affiliation, e-mail address of all protocol authors; provide physical mailing address of corresponding author	Pg. 1
Contributions	3b	Describe contributions of protocol authors and identify the guarantor of the review	Pg. 12
Amendments	4	If the protocol represents an amendment of a previously completed or published protocol, identify as such and list changes; otherwise, state plan for documenting important protocol amendments	N/A
Support:			
Sources	5a	Indicate sources of financial or other support for the review	Pg. 12/13
Sponsor	5b	Provide name for the review funder and/or sponsor	Pg. 12/13
Role of sponsor or funder	5c	Describe roles of funder(s), sponsor(s), and/or institution(s), if any, in developing the protocol	Pg. 12/13
**INTRODUCTION**			
Rationale	6	Describe the rationale for the review in the context of what is already known	Pg. 4-5
Objectives	7	Provide an explicit statement of the question(s) the review will address with reference to participants, interventions, comparators, and outcomes (PICO)	Pg. 5
**METHODS**			
Eligibility criteria	8	Specify the study characteristics (such as PICO, study design, setting, time frame) and report characteristics (such as years considered, language, publication status) to be used as criteria for eligibility for the review	Pg. 6-7

For peer review only

Information sources	9	Describe all intended information sources (such as electronic databases, contact with study authors, trial registers or other grey literature sources) with planned dates of coverage	Pg. 7-8
Search strategy	10	Present draft of search strategy to be used for at least one electronic database, including planned limits, such that it could be repeated	Supp. B
Study records:			
Data management	11a	Describe the mechanism(s) that will be used to manage records and data throughout the review	Pg. 8
Selection process	11b	State the process that will be used for selecting studies (such as two independent reviewers) through each phase of the review (that is, screening, eligibility and inclusion in meta-analysis)	Pg. 8
Data collection process	11c	Describe planned method of extracting data from reports (such as piloting forms, done independently, in duplicate), any processes for obtaining and confirming data from investigators	Pg. 8
Data items	12	List and define all variables for which data will be sought (such as PICO items, funding sources), any pre-planned data assumptions and simplifications	Pg. 9
Outcomes and prioritization	13	List and define all outcomes for which data will be sought, including prioritization of main and additional outcomes, with rationale	Pg. 9
Risk of bias in individual studies	14	Describe anticipated methods for assessing risk of bias of individual studies, including whether this will be done at the outcome or study level, or both; state how this information will be used in data synthesis	Pg. 9-10
Data synthesis	15a	Describe criteria under which study data will be quantitatively synthesised	Pg. 10-11
	15b	If data are appropriate for quantitative synthesis, describe planned summary measures, methods of handling data and methods of combining data from studies, including any planned exploration of consistency (such as I^2, Kendall's τ)	Pg. 10-11
	15c	Describe any proposed additional analyses (such as sensitivity or subgroup analyses, meta-regression)	Pg. 10-11
	15d	If quantitative synthesis is not appropriate, describe the type of summary planned	Pg. 11
Meta-bias(es)	16	Specify any planned assessment of meta-bias(es) (such as publication bias across studies, selective reporting within studies)	Pg. 11
Confidence in cumulative evidence	17	Describe how the strength of the body of evidence will be assessed (such as GRADE)	Pg. 11

*** It is strongly recommended that this checklist be read in conjunction with the PRISMA-P Explanation and Elaboration (cite when available) for important clarification on the items. Amendments to a review protocol should be tracked and dated. The copyright for PRISMA-P (including checklist) is held by the PRISMA-P Group and is distributed under a Creative Commons Attribution Licence 4.0.**

From: Shamseer L, Moher D, Clarke M, Ghersi D, Liberati A, Petticrew M, Shekelle P, Stewart L, PRISMA-P Group. Preferred reporting items for systematic review and meta-analysis protocols (PRISMA-P) 2015: elaboration and explanation. BMJ. 2015 Jan 2;349(jan02 1):g7647.

For peer review only

Supplement B: Search Strategy (Medline)

(1) Participants	1.	exp Overweight/ or exp Obesity/ or (adipos* or obes* or over?weight).tw
(2) Weight management intervention	2.	exp Body Weight/ or exp Life Style/ or exp Physical Activity/ or exp Obesity Management/ or exp Diet Therapy/ or exp Exercise/ or exp Diet/ or exp Behavior Therapy/ or exp Health Education/ or ((weight adj3 (body or chang* or los* or maint* or manage* or control* or reduc*)) or (body?mass?index or bmi) or (body adj3 mass) or life?style or (diet* or nutrition*) or (physic* adj3 (activ* or fit*)) or exercis* or (obes* adj3 (intervention or program* or camp* or treat*)) or (behavio?r* or psych*)).tw
(3) Mental health outcomes	3.	exp Behavioral Symptoms/ or exp Emotions/ or exp Mental Disorders/ or exp Adaptation, Psychological/ or exp Mental Health/ or exp Quality of Life/ or exp Self Concept/ OR (depress* or anxiet* or well?being or (quality?of?life or qol or health?status) or (affect* or mood*) or (health?related?quality?of?life or hrqol) or emotion* or (mental adj3 (health or well?being)) or (psych* adj3 (well?being or health)) or self?esteem or self?image or body?image or stress* or (emot* adj3 eating) or binge?eating).tw
(4) Study design	4.	exp Randomized Controlled Trials as Topic/ or randomized controlled trial.pt or controlled clinical trial.mp or randomi?ed.mp or randomly.mp or trial.mp
	5.	1 AND 2 AND 3 AND 4

Limit (5) to English-language results.

For peer review only

<u>Supplement C: Levels of evidence assessment</u>

Modified flow chart of the decision-making process for levels of evidence - based on study quality and study size. Consistent positive results (66.6% of relevant studies reporting significant positive results) are needed to achieve strong, moderate or limited levels of evidence.

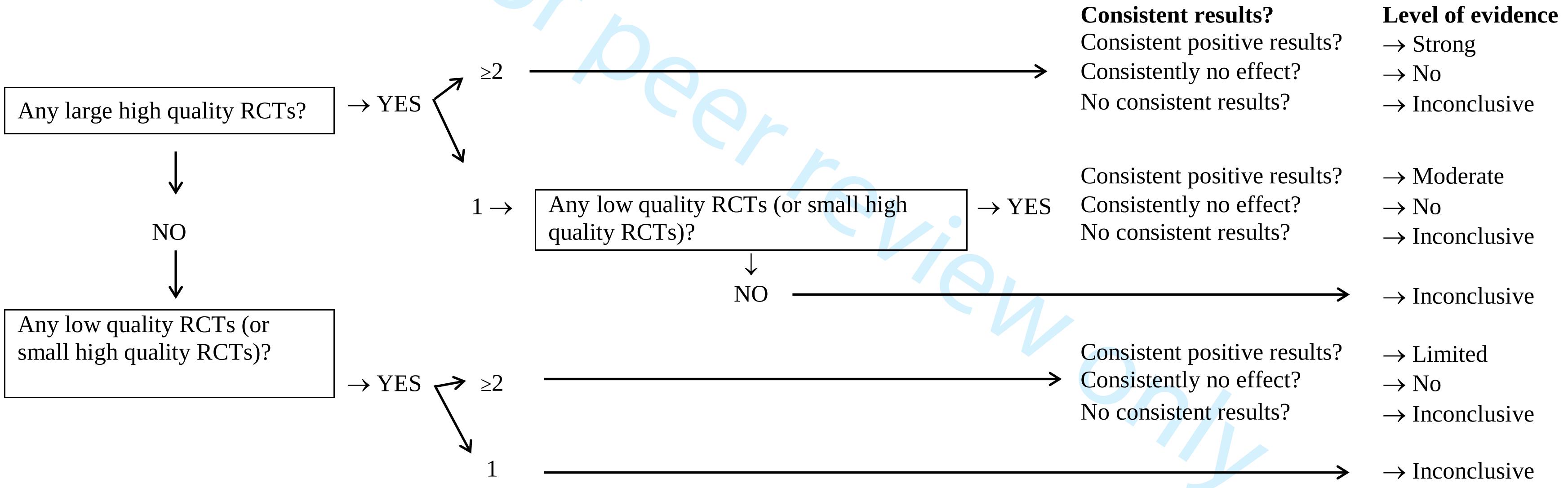

NOTE: studies including ≤ 250 participants or not providing sample size justifying a smaller sample size are considered ‘small’, studies including >250 participants are considered ‘large’.

For peer review only
